# The 6S‐model for person‐centred palliative care: A theoretical framework

**DOI:** 10.1111/nup.12334

**Published:** 2020-10-22

**Authors:** Jane Österlind, Ingela Henoch

**Affiliations:** ^1^ Department of Health Care Sciences Palliative Research Centre Ersta Sköndal Bräcke University College Stockholm Sweden; ^2^ The Sahlgrenska Academy Institute of Health and Care Sciences University of Gothenburg Gothenburg Sweden; ^3^ Angered Local Hospital Gothenburg Sweden

**Keywords:** nursing, nursing theory, palliative care, philosophy of nursing, practice, theory

## Abstract

Palliative care is provided at a certain timepoint, both in a person's life and in a societal context. What is considered to be a good death can therefore vary over time depending on prevailing social values and norms, and the person's own view and interpretation of life. This means that there are many interpretations of what a good death can actually mean for an individual. On a more general level, research in palliative care shows that individuals have basic common needs, for example physical, mental, social and spiritual well‐being. Therefore, in today's pluralistic Western society, it becomes important that palliative care is person centred to enable individuals to receive, as far as can be achieved, care that promotes as good a life as possible based on the person's own needs and preferences, and in accordance with evidence and current laws. For many years a research group, consisting nurse researchers together with nurses working in palliative care, has developed a model for person‐centred palliative care, the 6S‐model. The model's central concept is Self‐image, where the starting point is the patient as a person and their own experience of the situation. The other concepts: Self‐determination, Symptom relief, Social relationships, Synthesis and Strategies are all related to the patient's self‐image, and often to each other. The model's development, value base and starting assumptions are reported here, as are examples of how the model is applied in palliative care in Sweden. The model has been, and still is, constantly evolving in a collaboration between researchers and clinically active nurses, and in recent years also with patients and close relatives.

## 1 INTRODUCTION

### 1.1 A good death and the values in society

The perception of what is meant by a good death may vary depending on place and culture. Current norms and rituals about death in a society affect our approach to death and dying; this has been described by Walter (1994), Bauman (1994) and Kellehear (2007). Walter (1994) talks about three ideal types or constructions of death cultures: the traditional, the modern and the neomodern death. The traditional culture of death is described as religious, where faith was important and the priest was the authority that was sent for to give the dying person peace of mind. In the modern society, death moved from people's everyday lives to hospitals and institutions. The clinicians and medical science replaced the priest and religion as authorities. In the neomodern society, as in current society, the patient's autonomy and self‐determination are highly valued. The three approaches to death described by Walter (1994) can exist side by side, both within society and within the individual person, and can be of varying importance over time. The denial of dying, which makes the dying process invisible or even absent, is often described as a dominant societal discourse in the western world (Yalom, 1980). However, talking about death and dying, and about the dying person's will at the end of life, is not easy. Making death visible could ensure that there is room for life, also during the dying process. It is, therefore, crucial to acknowledge the person's own views of death and their preferences for care so that these can contribute to as good a death as possible for the individual. One way to have the possibility to get access to the person's own preferences is to use the 6S‐model, which has not earlier been presented as a whole to an international audience.

The aim of this paper is to describe the development of the 6S‐model for person‐centred palliative care and the theoretical assumptions of the model, and to give brief examples of its practical application in nursing.

### 1.2 Person‐centred care

Person‐centred care is an emerging concept and practice in health care, with an increasing number of theoretical and empirical papers being published on the topic (Byrne et al., 2020). There are similarities and differences in the descriptions of person‐centred care, but the main issue in the models is that care is co‐created by the person and the healthcare professionals, and that together they develop the care of the patient, sometimes called participation, partnership or co‐created care. This is based on the assumption that in encounters with healthcare professionals, a person is both a patient, which is a role they have or *something* that they are, and at the same time a person, which is who or *someone* that they are (Kristensson Uggla, 2014). In order for healthcare professionals to better understand a person's needs, the question of who they are becomes important. However, the theoretical and philosophical underpinnings for person‐centred care differ. Some refer to, for example, Paul Ricoeur (Ekman et al., 2011; Ohlen et al., 2017), some to Buber (Rushton & Edvardsson, 2018), others to Seyla Benhabib and John Rawls (Maatta et al., 2017), Gadamer (McCormack, 2003), or to existential oriented models such as Travelbee (1971). Person‐centred care has been analysed in the context of palliative care, and it has been suggested that person‐centred care should focus on both the suffering and the capability of the individual as a person (Ohlen et al., 2017).

The starting point of our model is to always see the patient as a person and, as such, someone who is a thinking, feeling, interpreting, social and creative being with the opportunity for lifelong development, even if the time ahead is limited (Cf. McCormack, 2003). In our understanding, person‐centred care is co‐created through the healthcare professionals engaging with the person in what Buber (1995) describes as a genuine dialogue in order to gain knowledge about how the person is experiencing his/her current situation. In practice, this means that the seriously ill person contributes with his/her experiences, knowledge, beliefs and preferences and the healthcare professionals contribute with their scientific knowledge and caring experiences.

### 1.3 Palliative care

One way of acknowledging the individual person's preferences for ways to achieve as good a death as possible is through palliative care that is based on the thoughts of Dame Cicely Saunders who was the initiator of the modern hospice movement. She described the person's narrative as essential for providing good care and achieving a good death (Saunders, 1978) A central concept, according to Saunders, was the term “total pain” (Clark, 1999; Saunders, 1978), where body and soul were regarded as inseparable. It was commonly desirable to strengthen both the patient's and the family's position in the care and promote the dying person's ability to live as meaningful a life as possible; these values are also found in the WHO definition of palliative care (WHO, 2002). A revised definition of palliative care, developed in a consensus process involving health care workers from countries in all income levels, states that palliative care “should be delivered based on need rather than prognosis, is applicable in all care settings and levels, and encompasses both general and specialist care” (Radbruch et al., 2020, p. 7). It also acknowledges the needs of persons in early phases of progressive diseases, such as heart failure or chronic obstructive pulmonary disease, who can benefit from palliative care and a caring approach that acknowledges the patient's symptoms, family, and thoughts about life and death.

### 1.4 Person‐centred palliative care

During the 1960–1980s, in the intersection between the modern and the neomodern views of death, the psychiatrist and thanatologist Avery D Weisman, an early proponent of person‐centred care, emphasized that it is only the individual person who can define what for them is a good death. Furthermore, he criticized the dualistic division of body and soul and acknowledged a holistic view of humans, which could contribute to the person being able to live as good a life as possible during the final phase of their life. Weisman used several terms to describe the meaning of a good death such as an appropriate death (Weisman, 1972), an acceptable death (Weisman & Kastenbaum, 1968) and a meaningful death (Weisman, 1988). Common to these terms is the starting point being the person with their own needs and preferences. This intention assumes that the person and the healthcare professionals engage in a dialogue, where the person is allowed to express what could contribute to meaning, dignity, relief from distress, and confirmation of their values and beliefs. The dialogue should be the basis for planning for both as good a life and as good a death as possible. This could be a life and a death which are in line with the person's own values and beliefs, a perspective that is emphasized in the 6S‐model developed in Sweden.

### 1.5 The 6S‐model for person‐centred palliative care

The 6S‐model for person‐centred palliative care is a method of integrating the palliative care definition (WHO, 2002), the palliative care philosophy as defined in the early hospice movement (Clark, 1999; Saunders, 1978), and the dialogue as described by Buber (1958, 1995) with Weisman's view that a person dies in the way he or she has lived (Weisman, 1972). Expressing one's beliefs, values and preferences for the final part of life could be a private topic, where there is a need for sensitivity and perception. The 6S‐model could be a guide to the initiation of a dialogue about this sensitive topic, but also a way to plan, provide, document and evaluate care. The 6S‐model for person‐centred palliative care acknowledges suffering in that the person is given the opportunity to express both good and bad experiences, and that the person will be the co‐creator of appropriate care.

**Figure 1:**
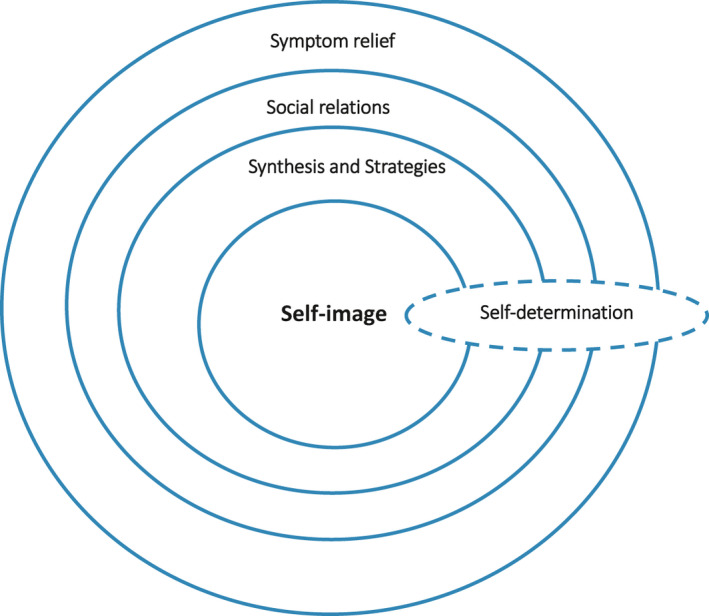
The relationships between the concepts in the 6S‐model

The model consists of six concepts: Self‐image, Symptom relief, Social relations, Synthesis, Strategies and Self‐determination. These concepts could be described as different dimensions of a holistic view of person and thereby represent different needs; they also refer to the concept of total pain (cf. Saunders, 1978). Self‐image is the core concept, mirroring a person's identity and referring to the fact that a person should have the possibility to preserve as good a view of self as possible, despite illness and dying. In order to preserve the self and Self‐image, and to live the best life possible, optimal Symptom relief is central. In the model, Symptom relief mainly elucidates physical suffering and physical care. Symptoms are a main reason for ill persons seeking health care, and it may be natural for them to talk about experiences of symptoms with healthcare professionals. Social relationships reflect the person's social needs, such as the need for fellowship with others. The Synthesis and choice of Strategies reflect the spiritual and existential needs. Having the opportunity to tell their own life story is essential, as is thinking about the meaning of life and what happens after death, which are concrete examples of issues related to the person's view and interpretation of life. Strategies could also include the choice of approaches for meeting their death, to the extent that such choices are possible. Self‐determination reflects the person's psychological needs, and the need to be an active participant in their life and to form the end of life according to their own beliefs and values. The person's Self‐determination needs to be acknowledged in encounters with healthcare professionals, in that the depth of dialogue and sharing of experiences needs to be determined by the ill person and their choice to not share experiences needs to be respected by the healthcare professionals. For the relationships between the concepts, see Figure 1.

## 2 DEVELOPMENT OF THE 6S‐MODEL

Hospice‐inspired care was first introduced in Sweden in the late 1970s and early 1980s, with both in‐patient hospice units and outpatient home‐care units. At one of these, the hospice Vitsippan that was started in 1991, the purpose was to provide good care and conduct patient‐related research to contribute to the further development of care. An important starting point was that the hospice should be based on a common philosophy of care and jointly formulated goals (Ternestedt, 1998). Together, staff and nurse researchers transformed the goals of the hospice philosophy into goals that would guide the meetings with the person and their close relatives. The central goal was to promote the person's autonomy and well‐being until their death. In addition, a new healthcare law was introduced in 1982 that emphasized the patient's self‐determination (Sahlberg Blom, 2001; SFS, 1982). Weisman and co‐workers’ description and operationalization of a good death became the starting point in the design of care. Weisman's definition of a good death included six criteria, formulated as six Cs: Care, Control, Composure, Communication, Continue and Closure (Weisman, 1974). Rinell Hermansson, (1990) transformed these into a Swedish healthcare context that has been continuously developed and refined over time (Rinell Hermansson, 1990). The 6S‐research group, consisting of nurse researchers, Professors, Associate Professors and PhDs labelled the concepts with words starting with S: Self‐image, Symptom relief, Social relations, Synthesis, Strategies and Self‐determination.

The purpose of the 6S‐model is to promote the person's opportunities to live the last period of their life, whether short or long, as well as possible, that is person‐centred palliative care. The core of the model was gradually developed to become Self‐image. This was perceived as logical because the model aimed at starting from the person's identity. When Self‐image was at the core, it became clear how the other S:s were dynamically related to Self‐image. The concepts together reflect the image of a good death (or appropriate death) and the importance of regarding the whole person in all areas of health care, particularly in vulnerable situations in early or late phases of palliative care as well as in conjunction with curative care interventions.

The development of the 6S‐model has taken place through annual 2‐day seminars in the 6S‐network, which is a collaboration between nurses working in units where the model has been applied and nurse researchers. Scientific papers have been published on parts of the model, on the 6S‐concepts, and also on their application which has been critically examined in relation to nurses’ clinical experience of their use (Carlander et al., 2011; Dwyer et al., 2011; Jeppsson & Thome, 2014; Ternestedt, 2009; Ternestedt et al., 2002), and a book about the whole model (Ternestedt et al., 2012, 2017). The model has been developed and adapted to the reality of palliative care in Sweden for almost 30 years. In recent years, the relevance of the concepts has also been tested in collaboration with patients acting as co‐researchers (Henoch & Osterlind, 2019). In addition, nurse researchers in collaboration with nurses working clinically in palliative care have developed a dialogue tool to promote the incorporation of the 6S‐concepts by nurses in clinical practice (Henoch & Osterlind, 2019). This might promote the co‐creation of care and help the person live as well as possible at the end of life.

## 3 ONTOLOGICAL, THEORETICAL AND EPISTEMOLOGICAL STARTING POINTS

### 3.1 Ontological starting points

The 6S‐model is based on a humanistic view where a person is seen as a thinking, knowing, social and meaningful being with opportunities for lifelong development (Erikson & Erikson, 1998). Human value is related to a person, his/her being and existence, and not just as an actor. Such a starting point becomes central to the care of people who are in a phase of life when physical and mental abilities decline and when they may have difficulty communicating their needs and wishes. Vulnerability can threaten the person's identity (Meleis, 2010). It requires both the will and the knowledge to try, as far as is possible, to understand the individual's needs when meeting with them. There is an ethical requirement in the relationship between the healthcare professionals and the patient, expressed by Løgstrup (1997), of holding another person's life in their hands.

### 3.2 Theoretical starting points

The essence of the 6S‐model concerns how the patient's identity or self‐image can be promoted in severe illness and near death. There are two theoretical perspectives which we have been particularly inspired by in the development of the 6S‐model, Erik and Joan Erikson's theory of human lifelong development (Erikson & Erikson, 1998) and Meleis’ (2010) theory of the transitions during life. These theories could be considered to complement each other in the view of people in crucial transitions, such as during the transition from healthy to severely ill or dying. Erikson and Erikson described at an early stage how the body, mind and society are interwoven, and the significance of human lifelong identity development. The identity or self is formed in the context in which the person lives. When a person is seriously ill and is about to die, care is an important context. This means that caregivers’ attitudes can be both encouraging and threatening for a patient's identity development. Erikson and Erikson's theory describes how relationships with others can both strengthen a person's self and well‐being as well as threaten it. An identity‐promoting approach should be applied by the staff to promote the person's influence over their own care and their remaining life.

During severe fatigue, impaired cognitive ability, and growing dependency there is a need for care that is based on the person's self and preferences. The proximity to death needs to influence caring encounters. Meleis’ (2010) description of the concept of transition and how this affects a person's identity experience makes this clear. She describes many events in life that affect a person's identity. Such transitions often mean that one's whole existence is threatened. Close to the final transition, the inequality in the relationship between patient and staff is highlighted and increases the demands on the staff. In the late phases of life, it is even more important that healthcare professionals listen to patients’ experiences, thoughts and feelings. Such silent and oral dialogues can contribute to an understanding of the patient's situation and provide an openness for a more equal relationship.

### 3.3 Epistemological starting points: the meeting and the dialogue

**Figure 2:**
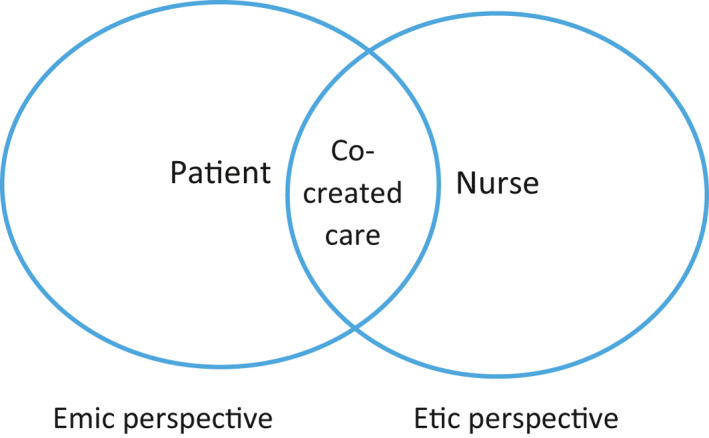
Description of emic and etic perspectives and co‐created care

As seen above, the theoretical assumptions of the 6S‐model are the identity of the person and the transitional journey the person is forced to embark on when approaching death. The transition concerns bodily and psychological changes, increased dependency and existential challenges, which could all be a threat to the person's identity. However, we also believe that a person has a developmental potential when approaching death (Erikson & Erikson, 1998). The epistemological challenge will, therefore, be how healthcare professionals should gain knowledge about who the person is, their self‐image, identity and crises, and the transitions they are experiencing, in order to pay attention to, respond to, and meet their needs. A dialogical approach, with interaction between the person and nurses, is a central starting point, and the prerequisite for person‐centred palliative care. The 6S‐model has its origin in the effort to pay attention to the person's own perception of an appropriate death and is based on theoretical and practical knowledge. The optimal idea of person‐centred care is that the patient and healthcare professionals together design and co‐create a care plan that promotes this type of care (Figure 2).

The encounter between the patient and the healthcare professional takes place in a situation where one part is the recipient of care and the other is the provider. The spiral of knowledge that the 6S‐concept constitutes in the meeting with the person could be a dialogue tool with the goal of reaching shared care (Shared decision‐making), where the asymmetry of the relationship (emic and etic perspective) is considered. The person has an inside perspective on his/her illness and health situation (emic perspective), unlike the caregiver who has an outside perspective (etic perspective). In other words, the ill person has a first‐person perspective and the healthcare professionals have a third‐person perspective and must try to understand from the outside what the person is telling them. When entering into a dialogue with the ill person and having a meeting in an I and Thou‐way as described by Buber (1958), the healthcare professionals will have the potential to gain a second‐person perspective of the patient. According to Buber, in a dialogue between one person and another, language is constituted and goes beyond the ‘I and you’ relationship. Healthcare professionals can, with the help of language, interact more closely with the person. However, it is important to realize that they can never have a first‐person perspective of the person. The healthcare professional cannot understand the person's overall situation but can only be aware that he/she always perceives the person based on his/her own understanding. This understanding will always have blind spots. The dialogue and how it is designed can be crucial to the person's opportunities to have as good a life as possible until their death.

## 4 EXAMPLES OF APPLICATIONS OF THE 6S‐MODEL

As mentioned earlier, the 6S‐model for person‐centred palliative care has its origin in the effort to give attention to the person's own perception of a good death and is based on theoretical and practical knowledge. The optimal idea of person‐centred care is that the patient and healthcare professionals together design and co‐create care that promotes this. The 6S‐model is based on a holistic view of the person and an approach including both cognitive and emotional components that acknowledge the person's values, beliefs and preferences. The model is also operationalized for care planning, documentation and continuous evaluation of health care. The approach includes recognizing the importance of integration of theoretical and experience‐based knowledge, which includes theoretical knowledge, practical professional knowledge and practical wisdom (Gustavsson, 1999). From the very beginning, the concepts of the 6S‐model have been developed based on research and clinical application by nurses in palliative care. Some practical applications of the 6S‐concepts based in research will be presented below. The questions that are used in the examples have been elaborated during later years in a collaboration between clinical working nurses, nurse researchers and patients and presented in a paper by Henoch and Osterlind (2019). The quotations in the text emanate from tape‐recorded interviews with nurses or from free‐text answers from nurses or patients to questionnaires using the questions. Under each of the concepts, the theoretical background that the concepts, questions and actions emanate from are presented.

### 4.1 Self‐image

The core concept in the model is Self‐image. Self‐image and identity are often used as synonyms and there are other concepts of equal importance such as self and personality. The basis of identity is the person and their own body.

Nurse: What do we need to know about you to be able to adapt your care in accordance with your needs and preferences?

Patient: I have a strong integrity and know what I want. I have general interests, and interests in technology and business development. I want healthcare professionals to listen to my needs and respect me for my age and experience.

From a care perspective, it is crucial and should be argued that healthcare professional does not need to know and should not know everything about a person's life, their successes and their failures. If the person wishes to tell their life story, then nurses need to be available for dialogue, and to listen and confirm. In a dialogue, the nurse could gain information about the person's important characteristics, beliefs and needs that should influence how the care is provided. In a study for the development of a tool for dialogue about the 6 S’s, it was found that an important issue for self‐image was maintaining everyday life (Henoch & Osterlind, 2019). After gaining knowledge about how everyday life could be maintained, the healthcare professionals can tailor the care to support the person in order to preserve self‐image and identity.

The theoretical underpinning of Self‐image is the theory of Erikson and Erikson (1998). They describe human development from a life‐cycle perspective, from birth to death. During each phase, the person must solve a specific crisis and find a kind of balance between the opposites of feelings that the crisis involves. From this crisis, a psychosocial force can then emerge. The time‐specific development phases described by Erikson and Erikson are as follows: infancy age, toddler age, age of play, school age, teens, early adulthood, adulthood and old age. The crises in these age phases are about finding ‘balance’ between the positive and the negative elements. Joan Erikson describes how a sense of despair may take over at the expense of the feeling of integrity, when impaired functions and increased dependence dominate everyday life. However, in accordance with Erikson and Erikson (1998) and Frankl (1962), the struggle can also transform into something bearable. Erikson and Erikson talk about the ability to find new strategies to relate to the changed body and self. To achieve a successful solution, for example, it is important that the feeling of confidence weighs heavier than the feeling of distrust. However, both components are important in developing a sense of hope. In the example above, the person mentions his/her integrity, which concerns one of the crises in old age, which could render a virtue of wisdom. A person's way of solving a crisis at a certain stage can develop during the next stage and throughout his/her life. An unresolved crisis, therefore, does not have to be permanent (Erikson & Erikson, 1998). The nurse's actions in the example above include taking into consideration the development phases; the nurse learns that this seems to be a person who would like to be given respect for his/her integrity, experiences and needs.

### 4.2 Symptom relief

In palliative care it is crucial to accomplish proper symptom relief and thereby give the prerequisites for preserving self‐image; the goal of symptom relief is that the dying person should be able to live as good a life as possible until death. Symptom relief alleviates the physical dimension of total pain and is part of the definition of palliative care (WHO, 2002).

Nurse: What do you find most distressing at present?

Patient: Never feeling safe, the symptoms come quickly and unexpectedly. The most distressing concern is chest pain, which comes at night. (Henoch & Osterlind, 2019)

Symptoms have been defined as ‘a person's subjective experience of changes in biopsychosocial function, sensory experience or understanding’ (Larson et al., 1994, p. 273). Symptoms are thus subjective experiences and are usually distinguished from signs that are objective manifestations of a disease that can be perceived by someone other than the person themself. Symptom experience includes frequency, intensity, discomfort and meaning. As described in Figure 1, a symptom could be an entrance into the person's values, beliefs and inner world. Difficult and unbearable symptoms are often a reason for a person seeking contact with health care. Therefore, a starting point for a dialogue and an important nursing action could be a discussion about symptoms. Having a dialogue about distressing symptoms is not unique in health care, especially not in palliative care, since the WHO definition stresses the importance of symptom relief (WHO, 2002). Nevertheless, in the 6S‐model, symptom relief is situated in a context where the physical symptoms are only a part of the care. Physical symptoms often have consequences for the person's life and could be an obstacle to social relationships, influence self‐image or self‐determination, hinder the person in dealing with existential challenges or, as in the example above, may result in the patient never feeling safe, instead feeling like a burden to others (Holmberg et al., 2019). In the example above, the nurse receives information about how symptoms create insecurity for the person, an aspect which needs to be acknowledged in their care.

### 4.3 Self‐determination

The right to autonomy or self‐determination is a basic principle of biomedical ethics (Beauchamp, 2013) and patient participation is of importance for the patient's ability to live a good life until death (WHO, 2002). How the patient understands and participates in symptom relief and their care is dependent on the patient's ability for self‐determination. For a person to be self‐determining, they must have enough information to be able to assess the consequences of their decisions. Both the ill person and the family have a need for collaboration with the healthcare professionals so that together they can design the last period of time as well as possible (Carlander, 2011; Carlander et al., 2011; Sahlberg Blom, 2001). A patient's self‐determination can thereby be crucial for their sense of self‐image.

Nurse: What is important for you to decide on in your care?

Patient: I want to be in control of my day myself, and manage myself if I can.

Nurse: If you do not decide for yourself, who will make decisions in your place?

Patient: My relatives and the healthcare staff that I know have the information they need. It has already been written down and the powers of attorney are in place.

At the end of life, the body is deteriorating and the person often becomes dependent on others, which in turn could influence self‐image (Holmberg et al., 2019; Schenell et al., 2019). If the person is not able to maintain control and self‐determination, decisions need to be handed over to others and then it is crucial that both the patient, the family and the healthcare professionals know who is going to be the proxy decision maker and whether that person is aware of the patient's preferences (Bollig et al., 2016; Sahlberg Blom, 2001). An important nursing issue is that the dialogue about this needs to be open and trustful in order to follow the person's preferences along the disease trajectory, and the person needs to be respected in their own decisions and choice of proxy decision maker (Henoch & Osterlind, 2019).

### 4.4 Social relationships

Self‐image can be maintained through the confirmation and feelings of being part of a social community. Receiving love and respect from others are important and can increase the opportunity to endure difficult situations (Yalom, 1980).

Nurse: Which people are especially important to you?

Patient: My four children. I have good relationships with them and we have an open communication about my illness.

Nurse: How can we make it easier for you to spend time with those you wish to?

Patient: I would like to have mobile oxygen equipment, so I can travel a little further and be away for half a day.

From the patient's perspective, during palliative care, relationships are often described as important in providing the opportunity to share a life‐threatening situation characterized by changes and losses in health status. Such changes, and an increased dependency on others, are examples of health‐related transitions (Schumacher & Meleis, 1994).

When inequality occurs, that is where the patient needs much more help than they can give back to close family members, they can feel like a burden. The experience of being a burden to others can lead to a reduced sense of dignity and feelings of guilt that affect self‐image and self‐determination and, by extension, affect the possibility of a good death. Mayeroff (1971) also describes the importance of trust in each other and that the reciprocity in a relationship can change from time to time, having significance for patients at the end of life. During certain periods of life, we are primarily givers of care and during other periods, we are recipients of care from others. Several issues related to social relationships are therefore important, such as family support, family acceptance of death and family preparation (Andershed & Ternestedt, 2001; Stajduhar et al., 2008). Here, the staff can be of great importance through being attentive, listening and confirming. It can be about simple things such as telling the patient that their hair looks good or they are wearing a nice shirt.

The interaction between patient, family members and healthcare professionals are central and the interaction is built on mutually shared knowledge and understanding about the situation (Andershed & Ternestedt, 2001). In the everyday life of palliative care, most decisions will also affect other people in the dying person's environment, such as their relatives and the healthcare professionals. The right of a person to be self‐determining and to achieve his or her own goals must not interfere with anyone else's self‐determination. People are basically dependent on others throughout their lives, although the degrees of dependency and self‐determination vary.

### 4.5 Synthesis

Synthesis and Strategies both concern the existential issue that can become more apparent closer to death. These S‐concepts are closely intertwined, where synthesis concerns summarizing one's own life retrospectively and reflecting over situations and experiences, while strategies are prospective and concerns the life remaining. The person is in a clear situational transition period, which can be described as a transition to something unknown (Schumacher & Meleis, 1994).

Nurse: What has been important in your life?

Patient: My life has been ever‐changing, both difficulties and joys, but the sum of them has been very good. I’ve had a long life!

Synthesis could concern existential issues, such as finding meaning in life (Frankl, 1962). Yalom (1980) has described four existential areas that are actualized at the end of life: death, loneliness, freedom, as well as meaninglessness and meaning. Death becomes apparent at the end of life and is a source of fear. Loneliness is also a source of fear and suffering. In the encounter with one's own death, many people experience what is commonly referred to as existential loneliness, which is difficult or impossible to completely share with others (Bolmsjo et al., 2002; Sand & Strang, 2006). Freedom concerns a person being often obliged to choose between different options and having to take responsibility for these choices. At the end of life, a person wishes to find meanings and patterns in life and suffers when finding meaninglessness (Yalom, 1980). From a salutogenic perspective, sociologist and researcher Aaron Antonovsky have developed the theory of sense of coherence (Antonovsky, 1987); to experience a sense of coherence, an event must be comprehensible, manageable and meaningful to the person. Talking about one's life means creating a story, which then grows into the story itself. This means that the life story changes depending on how it grows, the response the narrator receives and the situation in which the story is told. Synthesis is a way of understanding and concluding about how life has been, as in the example above. When attentively listening to the person's life story, healthcare professionals can contribute to the person's understanding and help them find meaning in life and illness.

### 4.6 Strategies

The perception of one's imminent death means a confrontation with what is commonly termed an existential border situation (Grieder, 2009) or living in no‐man's land (Ekwall et al., 2007). It is also close to the concept described as existential homelessness (Molony, 2010; Rasmussen et al., 2000), that is not feeling at home in one's self or one's situation, which can affect both the person's life and how the person values the content of life.

Nurse: What do you think about the time ahead?

Patient: I know more or less how the cancer will develop. But I don't know how the pain will be, I hope that there is effective pain relief. What I hope for is to celebrate another Christmas and to experience our 60th wedding anniversary.

Weisman (1986) and Erikson and Erikson (1998), argue that a person can reach a sense of life's completion. However, consent or acceptance does not mean that the last period of life is free from anxiety or hovering between feelings of hope and despair (Feigenberg, 1980; Qvarnström, 1978). Many ill people have hopes for the future, which may be participating in a family celebration (Benzein et al., 2001) or the hope of being cured. This hope has been called the hope for the ultimate rescuer (Yalom, 1980). However, hope can also concern the patient living and feeling a sense of confidence that what must happen will happen, a kind of reconciliation with both life and death (Benzein et al., 2001). Through listening sensitively to the person's story, healthcare professionals may help the person to promote a sense of coherence, that is synthesis, self‐image and identity in the encounter with death. This could be the basis for strategies about what the person wishes to accomplish before death.

### 4.7 The 6S‐model in a societal perspective

Achieving person‐centred care in the daily work of healthcare professionals requires resources at different levels. At a micro level, the staff need knowledge, understanding and person‐centred guidance. One limitation of the model is that it could be regarded as a checklist or solely as a documentation system labelled as person‐centred care. On the contrary, the model should be used as a care approach that requires healthcare professionals to be responsive to the patient's verbal and non‐verbal needs. Buber (1958) describes the need for sensitivity and maturity in order to be able to provide personal guidance and reach a dialogue. At the meso level, this means that the care culture is required to support such approaches. Supportive actions include, for example, that managers in healthcare support the approach of the 6S‐model. This means that healthcare professionals are given the opportunity for regular training in communication skills, the opportunity for clinical reflection, and work schedules that allow continuity of care and give them the potential to adapt the care of each person to the values, beliefs and needs of that person.

At a macro level, a good death could be regarded in the traditional, modern or neomodern way (Bauman, 1994; Walter, 1994), but the importance is in making resources available to allow the promotion of person‐centred palliative care in a more long‐term perspective. Being able to develop a care culture in this direction that is sustainable requires willingness and a capacity for change and development, and that organizational, financial and human resources are allocated for this at all three levels.

## 5 CONCLUSION

Although the 6S‐model is a practical model developed in close collaboration with registered nurses working in palliative care units, the concepts and the model as a whole are based on palliative care philosophy (Clark, 1999; Saunders, 1978; WHO, 2002), the Erikson developmental theory (Erikson & Erikson, 1998), and the dialogue described by Buber (1958, 1995). The dialogue between the ill person and the healthcare professionals is central to the model; no quality palliative care can take place without such a dialogue. Schumacher and Meleis (1994) and Meleis et al. (2000) also argue that the dialogue is essential at times of vulnerability and during transition, such as development and transitions from healthy to ill, since the changes themselves constitute a threat to self‐image and identity. Giving the ill person the opportunity to be a co‐creator of their care increases the possibility that the care is adapted to the person's values, beliefs, and preferences and the care is person‐centred.

## CONFLICT OF INTEREST

None declared for any authors.
